# Low *PKCa* expression within the MRD-HR stratum defines a new subgroup of childhood T-ALL with very poor outcome

**DOI:** 10.18632/oncotarget.2062

**Published:** 2014-06-06

**Authors:** Gloria Milani, Paola Rebora, Benedetta Accordi, Luisa Galla, Silvia Bresolin, Gianni Cazzaniga, Barbara Buldini, Rossella Mura, Saverio Ladogana, Eugenia Giraldi, Valentino Conter, Geertruy Te Kronnie, Maria Grazia Valsecchi, Giuseppe Basso

**Affiliations:** ^1^ Laboratory of Oncohematology, Department of Womens and Children's Health, University of Padova, Italy; ^2^ Center of Biostatistics for Clinical Epidemiology, Department of Health Sciences, University of Milano-Bicocca, Monza, Italy; ^3^ Centro Ricerca Tettamanti, University of Milano-Bicocca, Ospedale San Gerardo, Monza, Italy; ^4^ Oncoematologia Pediatrica e Patologia della Coagulazione, Ospedale Regionale per le Microcitemie, Cagliari, Italy; ^5^ Oncoematologia Pediatrica, Ospedale “Casa Solievo della Sofferenza”, San Giovanni Rotondo, Italy; ^6^ U.O. Pediatria, Ospedale Papa Giovanni XXIII, Bergamo, Italy; ^7^ Pediatric Department, University of Milano-Bicocca, Ospedale San Gerardo, Monza, Italy

**Keywords:** Low PKCα expression, Childhood T-cell Acute Lymphoblastic Leukemia, T-ALL prognostic marker

## Abstract

Pediatric T-cell Acute Lymphoblastic Leukemia (T-ALL) outcome has improved in the last decades, yet one patient in every four still relapses. Except treatment response and immunophenotype, few markers are reliably prognostic in pediatric T-ALL patients. Aiming to improve T-ALL risk stratification, we investigated a new candidate biomarker with potential prognostic relevance. A phosphoproteomic screening of 98 pediatric T-ALL samples at diagnosis had been performed using the high-throughput Reverse Phase Protein Arrays technique, which led to the identification of PKCαS657 as an activated protein with a broad variation among T-ALL samples. To evaluate PKCα potential as a prognostic biomarker, *PKCα* expression was analyzed using RQ-PCR in a cohort of 173 patients, representative of ALL2000-ALLR2006 AIEOP study. A threshold of *PKCα* expression with the highest discrimination for incidence of relapse was identified. Patients with *PKCα* down-regulation, compared to patients with *PKCα* levels above the threshold, presented a markedly increased cumulative incidence of relapse (43.8% vs. 10.9%, P<0.001), as well as a worse 4-year overall survival (66% vs. 87.9%, P=0.002) and event-free survival (53.1% vs. 85.2%, P=0.002). In particular, low *PKCα* expression identified cases with extremely poor outcome within the high-risk minimal residual disease (MRD) stratum, their incidence of relapse being of 69% vs. 15% in the high *PKCα* levels group. In a multivariate analysis adjusting for main prognostic features, *PKCα* proved to be an independent prognostic factor related to incidence of relapse. Very high risk patients within the high-risk MRD stratum, identified by *PKCα* expression, could be proposed for experimental therapeutic protocols.

## INTRODUCTION

Pediatric T-cell acute lymphoblastic leukemia (T-ALL) represents 10–15% of pediatric ALL. The process leading to the malignant transformation of immature T-cells has been described as a multistep progression, involving several genomic aberrations altering the normal control of T-cell development and proliferation. Although T-ALL pathogenesis has been extensively studied, the pivotal mechanisms involved in T-ALL progression and relapse are still mostly unexplored [1-8]. Current intensified treatment protocols have achieved 5-year relapse-free survival rates of about 75%. Nevertheless, about 25% of T-ALL patients remain at high risk of early relapse [5, 9]. To date, T-ALL pediatric patients have been uniformly treated with intensive chemotherapy in all major protocols and patient stratification has been primarily based on WBC count, early response to therapy, such as to glucocorticoid prophase therapy, early achievement of complete remission (CR) and on minimal residual disease (MRD) findings [10]. Recently, early T-cell precursor acute lymphoblastic leukemia (ETP-ALL) was identified by immunophenotype as a subgroup characterized by a very poor outcome and treatment failure [11]. The identification of additional prognostic markers in childhood T-ALL is mandatory to improve risk stratification and thus spare low-risk patients from the toxic side effects of aggressive therapeutic approaches as well as identify patients with a very poor prognosis who might benefit from new treatments.

In the current study, a phosphoproteomic screening of 98 pediatric T-ALL samples at diagnosis has been performed by means of the high-throughput Reverse Phase Protein Arrays (RPPA) technique, which led to the identification of PKCαS657 as an activated protein characterized by a broad variation in T-ALL. We observed that low PKCαS657 activation resulted to be concordant with PKCα protein expression. Analyzing a larger cohort of 173 T-ALL patients, a broad variation in *PKCα* gene expression was observed as well. To evaluate *PKCα* potential as a prognostic biomarker, we investigated the association of *PKCα* mRNA expression levels with prognosis in a representative cohort of pediatric T-ALL patients treated in AIEOP (Associazione Italiana Ematologia Oncologia Pediatrica) centers. We showed that *PKC*α down-regulation in T-ALL patients is associated with both a high incidence of relapse and a worse survival. Within the MRD-HR stratum, we identified a new very high risk subgroup of T-ALL patients characterized by low *PKCα* expression levels.

## RESULTS

### PKCαS657 broad variation among T-ALL patients

We retrospectively analyzed 98 T-ALL pediatric patients at diagnosis, enrolled in AIEOP-BFM treatment protocols, using Reverse Phase Protein Arrays (RPPA) (Supplemental Material, Table S1). Expression or activation of 53 proteins belonging to different signaling pathways regulating proliferation, apoptosis and cell cycle, were analyzed (Supplemental Material, Table S2). Amid all proteins studied, PKCαS657 showed a broad variation among patients, with a continuous distribution among the analyzed samples (Supplemental Material, Figure S1A).

We validated PKCαS657 activation by Western Blot and we also studied PKCα total protein. The low activation of PKCαS657 observed in T-ALL patients by RPPA resulted to be in parallel with PKCα protein expression levels (Supplemental Material, Figure S1B), which is likely to be due to a reduced *PKCα* gene expression.

### *PKCα* mRNA expression at diagnosis and risk of relapse

*PKCα* gene expression was studied by RQ-PCR in 173 T-ALL patients at diagnosis, based on RNA sample availability. The set of 173 samples was representative of all T-ALL patients enrolled in the treatment protocol AIEOP-BFM ALL2000 and ALLR2006 with a very similar outcome in terms of EFS and CIR (Supplemental Material, Figure S2). Table 1 summarizes the characteristics of the group of patients. PKCα mRNA levels also revealed a broad distribution among T-ALL patients studied by RQ-PCR (Supplemental Material, Figure S3). In the first place, in order to investigate the association between *PKCα* mRNA and patient outcome, CIR was estimated in four groups, defined by the quartiles for *PKCα* mRNA expression values. The highest incidence of relapse was found in patients with the lowest *PKCα* expression at diagnosis (lower than the 25th percentile), whereas CIR decreased along with increasing levels of *PKCα* (Supplemental Material, Figure S4).

**Table 1:** Clinical and biological features of T-ALL patients enrolled in AIEOP-BFM ALL2000-ALLR2006 protocol and included in the study

Characteristics	Patients (N=173)	
	N	%
**Sex**		
Female	32	18.5
Male	141	81.5
**Age**		
<5 y	59	34.1
6–9 y	51	29.5
10–17 y	63	36.4
Immunophenotype*		
Early T	66	38.2
Cortical	71	41.0
T mature	26	15.0
BICLON T	3	1.7
T	7	4.1
**WBC count (/mm^3^)**		
≤50000	56	32.7
>50000	115	67.3
Unknown	2	
**Prednisone response (circulating blasts/μL on day 8)**		
Good (<1000)	102	63.4
Poor (≥1000)	59	36.6
Unknown	12	
**MRD**		
MRD-SR	19	14.3
MRD-IR	85	63.9
MRD-HR	29	21.8
Unknown	40	
**Risk group (final stratification)**		
SR	18	10.4
IR	83	48.0
HR	72	41.6
**DNA index**		
0.8–<1	2	1.3
1–<1.16	152	96.8
1.16–1.6	2	1.3
≥1.6	1	0.6
NE	16	
**CNS**		
Positive	14	8.4
Negative	152	91.6
Unknown	7	
**t(4;11)**		
Negative	156	100
Unknown	1	
**t(9;22)**		
Positive	1	0.6
Negative	155	99.4
Unknown	17	
**PKCα**		
Median (1^st^–3^rd^ quartiles)	0.202 (0.037–0.615) 0.58	

* Early T-cell Precursors (ETP) T-ALL diagnosis was not performed for the ALL2000-ALLR2006 treatment protocol study.

Abbreviations: CNS, central nervous system; HR, high risk; IR, intermediate risk; MRD, minimal residual disease; NE, not evaluated; SR, standard risk; WBC, white blood cell.

### *PKCα* mRNA expression at diagnosis as a prognostic biomarker: definition of a threshold

**FIGURE 1:**
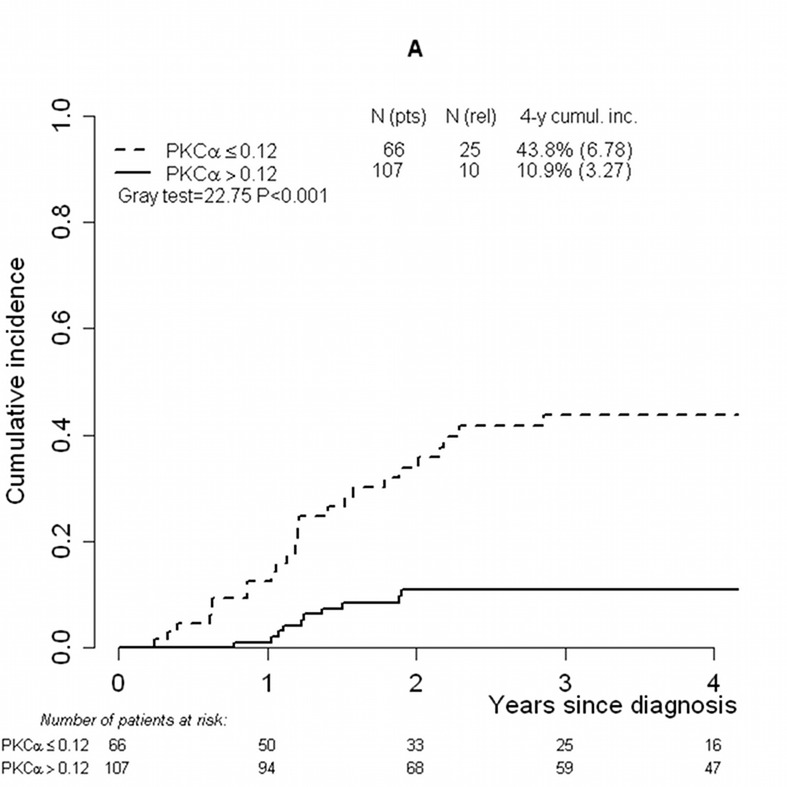

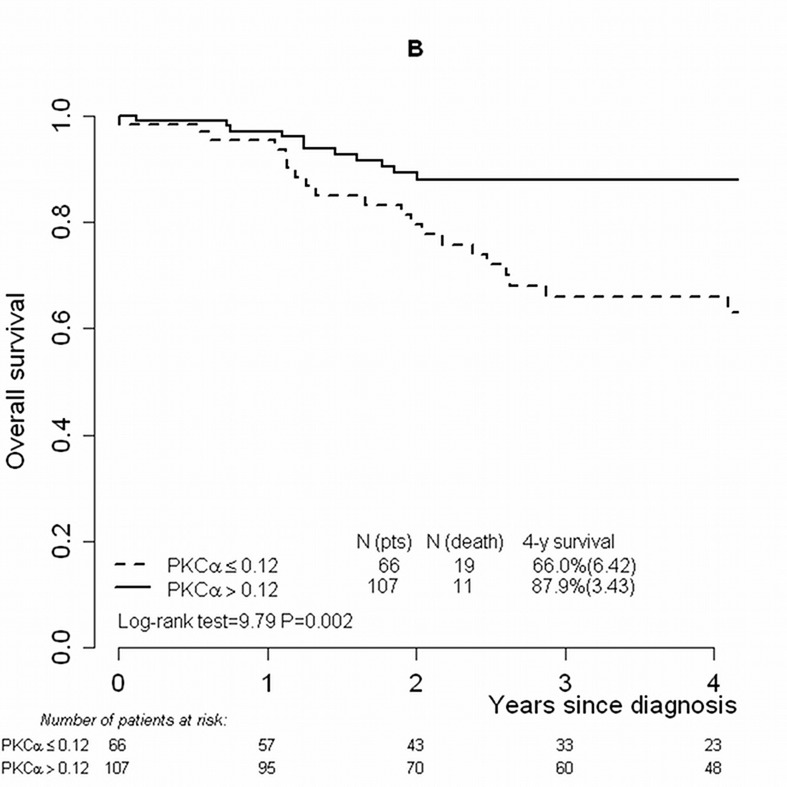

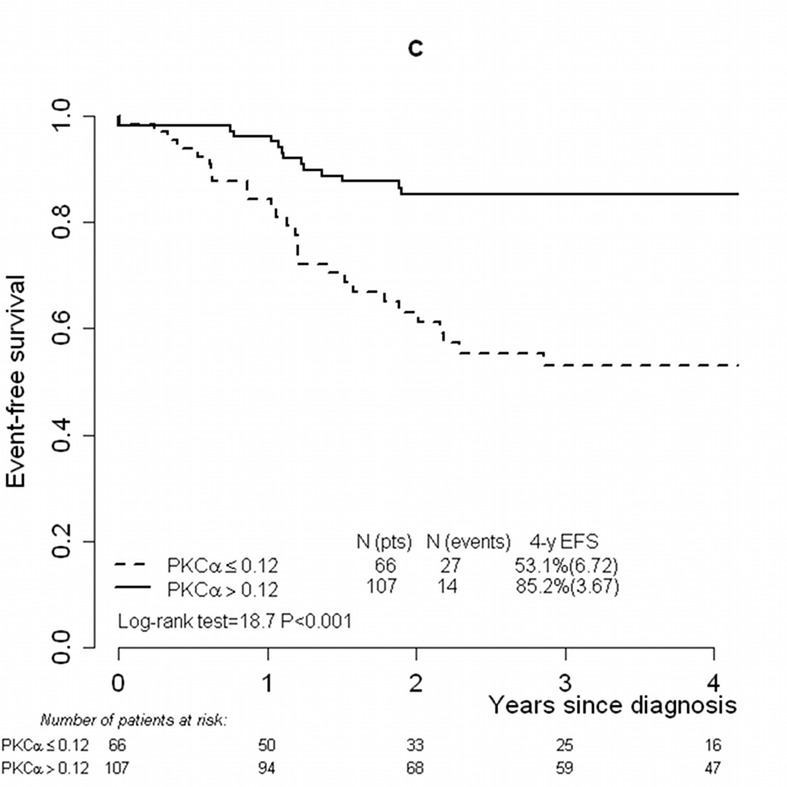
Cumulative incidence of relapse, overall survival and event-free survival analyses performed considering the threshold (0.12) defined on *PKCα* mRNA expression values Cumulative incidence of relapse in T-ALL patients studied by RQ-PCR and categorized by means of the defined threshold (0.12) (A). Overall survival (B) and event-free survival (C) analyses in the T-ALL cohort categorized by means of the threshold. Abbreviations: Cumul.inc., cumulative incidence; EFS, event-free survival; pts, patients; rel, relapse; RQ-PCR, real-time quantitative PCR; T-ALL, T-cell acute lymphoblastic leukemia.

In the second place, to find the optimal threshold in *PKCα* expression discriminating between patients with higher and lower risk of relapse, the ROC curve (maximum Youden index) was used. The cut-off was found to be 0.12, with 71.2% (95% confidence interval [CI]: 54.5%–83.6%) sensitivity and 72.4% (95%CI 63.7%–79.6%) specificity (results are similar if other statistics in the ROC plane are used; data not shown). Application of the calculated threshold categorized T-ALL patients into two groups with a significant difference in the incidence of relapse: 4-year CIR was 43.8% (standard error [SE] 6.78) and 10.9% (SE 3.27) in patients whose *PKCα* expression values were lower and higher than 0.12, respectively (P<0.001) (Figure 1A). OS and EFS were also significantly different (P=0.002 and P<0.001, respectively): patients with *PKCα* expression lower than 0.12 presented a worse OS and EFS (4-yr OS: 66%, SE 6.42 and 4-yr EFS: 53.1%, SE 6.72) in comparison with those whose *PKCα* expression was higher than 0.12 (OS: 87.9%, SE 3.43 and 4-yr EFS: 85.2%, SE 3.67) (Figure 1B and 1C).

### Association between *PKCα* expression and T-ALL prognostic factors

The association between *PKCα* expression and the main biological and clinical features of the patients (i.e. sex, age, immunophenotype, WBC count, prednisone response, MRD strata, central nervous system invasion and final stratification) was evaluated by means of Chi-square test and a significant relation between *PKCα* expression and both prednisone response and MRD strata was found (Table 2). As to steroid response, 61% of the patients included in the PPR group showed low *PKCα* mRNA levels while 39% presented high *PKCα* levels. With respect to the PGR group, 26% of the cases revealed low *PKCα* whereas 74% showed high *PKCα* (P<0.0001). Moreover, low *PKCα* was found in 55.2% of the MRD-HR patients vs. 44.8% of the cases presenting high *PKCα* levels. 29.4% of patients showed low *PKCα* mRNA in the MRD-IR stratum vs. 70.6% of the cases with high *PKCα*. Low *PKCα* was reported only in 15.8% of patients included in the MRD-SR class compared to 84.2% of the cases with high *PKCα* (P=0.009).

**Table 2:** Association between *PKCα* expression and clinical and biological features of T-ALL patients

	PKCα ≤ 0.12		PKCα > 0.12				
Characteristics	N(pts)	%	N(pts)	%	Total N(pts)	Chi-square	P value
**Sex**							
Female	8	25.0	24	75.0	32	2.8775	0.0898
Male	58	41.1	83	58.9	141		
**Age**							
<5 y	25	42.4	34	57.6	59	0.6942	0.7067
6–9 y	18	35.3	33	64.7	51		
10–17 y	23	36.5	40	63.5	63		
**Immunophenotype Early T**							
Early T	30	45.4	36	54.6	66	2.4128	0.1203
Other	36	33.6	71	66.4	107		
**WBC count (/mm^3^)**							
≤50000	18	32.1	38	67.9	56	1.2172	0.2699
>50000	47	40.9	68	59.1	115		
Unknown	1		1		2		
**Prednisone response (circulating blasts/μL on day 8)**							
Good (<1000)	27	26.5	75	73.5	102	18.7291	<0.0001
Poor (≥1000)	36	61.0	23	39.0	59		
Unknown	3		9		12		
**MRD**							
MRD-SR	3	15.8	16	84.2	19	9.4761	0.0088
MRD-IR	25	29.4	60	70.6	85		
MRD-HR	16	55.2	13	44.8	29		
Unknown	22		18		40		
**Risk group (final stratification)**							
SR	3	16.7	15	83.3	18	21.7583	<0.0001
IR	21	25.3	62	74.7	83		
HR	42	58.3	30	41.7	72		
CNS							
Positive	4	28.6	10	71.4	14	0.5713	0.4497
Negative	59	38.8	93	61.2	152		
Unknown	3		4		7		

Abbreviations: CNS, central nervous system; HR, high risk; IR, intermediate risk; MRD, minimal residual disease; pts, patients; SR, standard risk; WBC, white blood cell.

### Prognostic impact of *PKCα* expression on relapse event

Multivariate analysis showed a prognostic significant role of *PKCα* mRNA expression in the hazard of relapse, with a significant 3-fold increase in the rate of relapse in patients whose *PKCα* mRNA levels are lower than the defined threshold in comparison with those presenting *PKCα* mRNA levels above it (P=0.004; Table 3). The model accounted for the major known prognostic features (final risk stratification, age, WBC count, immunophenotype). Early T immunophenotype was related to a 4-fold increase in relapse compared to other phenotypes (P<0.001). The HR subgroup for final stratification was also associated with a significantly higher risk of relapse (hazard ratio=3.56 for HR vs. IR+SR patients, P=0.003), along with WBC count at diagnosis.

**Table 3:** Multivariate analysis of relapse occurrence in 171 T-ALL patients

Characteristics	Hazard Ratio (95%CI)	p value
*PKCα* expression ≤0.12 vs >0.12	3.09 (1.43–6.67)	0.004
Risk group HR vs IR+SR	3.56 (1.53–8.27)	0.003
Age ≥10 vs <10 years	1.81 (0.93–3.52)	0.080
WBC >50000 vs ≤50000	2.57 (1.04–6.37)	0.041
Immunophenotype (Early T vs other)	3.98 (1.97–8.04)	<0.001
N_patients_=171; N_relapse–events_=36		

PKCα mRNA expression categorized on the basis of the calculated threshold; patient AIEOP final stratification (HR vs IR+SR), age at diagnosis (≥10 vs. <10 years), WBC count at diagnosis (>50000/mm^3^, ≤50000/mm^3^), and immunophenotype (early T vs. others) were included in the analysis. Abbreviations: CI, confidence interval; HR, high risk; IR, intermediate risk; SR, standard risk; T-ALL, T-cell acute lymphoblastic leukemia; WBC, white blood cell.

### *PKCα* expression identifies very high risk patients within the MRD-HR stratum

**FIGURE 2:**
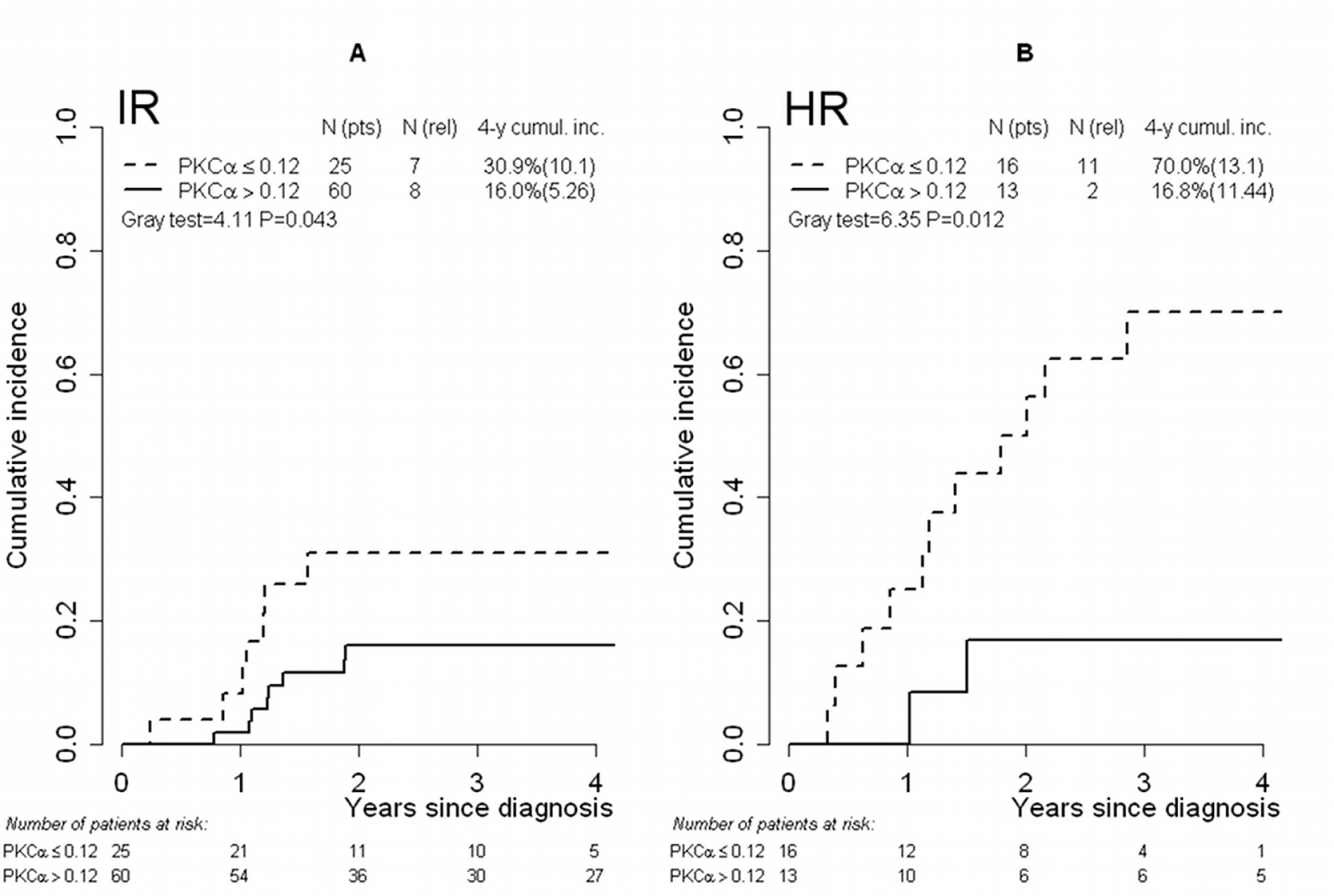
Cumulative incidence of relapse in risk groups defined by MRD level T-ALL IR group of patients (A) and HR group of patients (B) categorized by means of the threshold (0.12) defined on *PKCα* mRNA expression values. Abbreviations: Cumul.inc., cumulative incidence; HR, high risk; IR, intermediate risk; MRD, minimal residual disease; pts, patients; rel, relapse; T-ALL, T-cell acute lymphoblastic leukemia.

We analyzed the prognostic value of *PKCα* expression separately within the MRD-HR subgroup (N=29 patients) as well. The defined *PKCα* threshold identified 16 patients with low *PKCα* expression and 13 patients with high *PKCα* expression, of whom 69% and 15% respectively experienced relapse (Table 4). Also patients classified in the MRD-IR group presented a higher percentage of relapse in the low *PKCα* expression group (32% vs. 13% in the low and high *PKCα* mRNA groups, respectively). With regard to incidence of relapse, this analysis shows that the discriminating ability of the *PKCα* threshold is consistent within MRD based subgroups (P=0.012 and P=0.043 for CIR comparison in HR and IR group respectively, Figure 2).

**Table 4:** Relapse distribution in MRD classes categorizing T-ALL patients on PKCα mRNA expression (≤0.12 and >0.12)

Relapse distribution on PKCα mRNA expression in relation to MRD stratification (N_patients_ =133)						
*PKCα* mRNA expression	MRD					
	**Standard risk** (MRD-SR) N=19		**Intermediate risk** (MRD-IR) N=85		**High risk** (MRD-HR) N=29	
	N(rel)/N(pts)	% relapse	N(rel)/N(pts)	% relapse	N(rel)/N(pts)	% relapse
>0.12	0/16	0	8/60	13.3	2/13	15.4
≤0.12	0/3	0	8/25	32.0	11/16	68.8

Abbreviations: HR, high risk; IR, intermediate risk; MRD, minimal residual disease; pts, patients; rel, relapses; SR, standard risk; T-ALL, T-cell acute lymphoblastic leukemia.

To further corroborate the prognostic value of *PKCα* expression levels in patients belonging to MRD-HR stratum, we compared PKCαS657 activation levels of patients that relapsed (N=9) with those of patients that remained in CR (N=10), belonging to MRD-HR group. Analyses of RPPA data showed a significant difference in PKCαS657 activation levels between the groups of patients (P=0.035). Indeed, patients that relapsed presented a low PKCαS65 protein activation whereas patients in CR showed high PKCαS65 levels at diagnosis.

The identification of very high risk T-ALL patients within the MRD-HR stratum, discriminated by *PKCα* low expression, is of particular clinical relevance since no other prognostic factors are able to recognize this subgroup of patients characterized by an unfavourable outcome.

### Gene expression analyses on very high-risk subgroup within MRD-HR T-ALL patients

In order to investigate the molecular signature related to *PKCα* expression levels within the MRD- HR subgroup, gene expression analyses were performed on 13 T-ALL samples belonging to this group, previously studied by RQ-PCR, by the means of HG-U133 Plus 2.0 arrays on Affymetrix platform. Among this subgroup, 9 samples showed *PKCα* expression lower than the defined threshold whereas 4 samples presented *PKCα* expression above the threshold. Heatmap reported in Figure S5 (Supplemental Material) shows the 50 most differentially expressed genes between these two subgroups. Of note, also *PKCα* resulted differently expressed among the 50 top genes.

Using Gene Set Enrichment Analysis (GSEA), gene expression profiles of these two groups, categorized on the defined threshold, were examined for different expression patterns. Negative enrichment score indicated correlation with the low *PKCα* expression MRD-HR group. Among the GSEA molecular signature databases considered, this subgroup resulted to be enriched in several gene sets with FDR<0.05 such as those involved in cell cycle, telomerase maintenance and RNA transcription (Supplemental Material, Table S3, S4 and S5; Figure S6). Remarkably, also considering gene set C4 database, the cancer gene neighbourhood GSEA MsigDB database [12], the low *PKCα* expression MRD-HR group resulted enriched in gene modules related to cell cycle regulation, transcription, establishment and maintenance of chromatin architecture and chromosome stability (FDR<0.05).

## DISCUSSION

In the last few decades, the survival in pediatric T-ALL patients has significantly improved, yet one patient in every four still encounters relapse, in particular early relapse [5, 7, 9, 13]. To date, with the exception of subgroups defined by MRD and immunophenotypes, few biomarkers have proved to be reliable in predicting T-ALL patient prognosis. MRD analysis is nowadays considered the most powerful independent prognostic factor, improving long-term outcome prediction for T-ALL, but it allows patient risk stratification only after the induction phase of therapy. The introduction of new prognostic factors that can lead to a more accurate patient risk stratification and the related treatment approach is expected to improve T-ALL outcome. Moreover, the early identification of patients with increased risk of relapse, also within already defined MRD strata, underscores the need to develop new therapeutic approaches for high risk patients, while sparing others from undue toxic therapies.

In this study, we have identified *PKCα* as a new prognostic marker in pediatric T-ALL and we have defined its prognostic impact. In the first place, by means of a phosphoproteomic approach, we identified PKCαS657 as an activated protein with a broad variation among T-ALL. PKCα is a protein kinase expressed in many tissues and associated with a number of biological functions such as cell proliferation, differentiation, cell cycle control, apoptosis, cell survival, cell adhesion and cell motility. Cellular responses to activated PKCα depend on its temporal activation, downstream targeted pathways, and tissue specificity. In cancer, PKCα has been described as a tumor suppressor as well as a protein with an oncogenic role [14, 15]. In particular, PKCα deficiency or down-regulation has been reported in pancreatic cancer cells as well as in intestinal tumorigenesis [16, 17]. Moreover, the expression of a dominant-negative PKCα in murine fetal liver–derived hematopoietic progenitor cells resulted in enhanced proliferative capacity and in the generation of a B-cell chronic lymphocytic leukemia–like population *in vitro* [18]. On the other hand, *PKCα* overexpression has been associated with poor prognosis in gastric carcinoma, breast cancer, hepatocarcinoma and pancreatic cancer [19-24].

After identifying of PKCαS657 as a protein kinase with a broad variation among T-ALL patients, we observed that low PKCα activation resulted to be concordant with total PKCα protein expression. Therefore, we proposed PKCαS657 as a candidate biomarker and we investigated in depth its potential association with T-ALL prognosis. *PKCα* mRNA expression was analyzed in a cohort of 173 T-ALL pediatric patients at diagnosis, representative of the AIEOP-BFM ALL2000-ALLR2006 treatment protocol. In this larger cohort, a broad variation in *PKCα* gene expression was observed as well.

A higher incidence of relapse in the group with low *PKCα* mRNA expression values was found in comparison to patients with high *PKCα* expression (4-year relapse cumulative incidence 43.8% vs. 10.9%, respectively). *PKCα* thus proved to be a relevant prognostic biomarker associated with incidence of relapse in T-ALL patients. According to the optimal threshold analysis based on the ROC curve extended to deal with censored data, two subgroups of T-ALL patients were defined by the threshold of 0.12. The two subgroups showed different incidence of relapse, OS and EFS. Our results emphasize the clinical relevance of the threshold defined on *PKCα* mRNA expression at diagnosis as a new approach to discriminate T-ALL prognosis. In the multivariate analysis that accounted for the most relevant prognostic factors already considered in clinical practice, patients whose *PKCα* mRNA levels were lower than the defined threshold presented a significant 3-fold increase in relapse rate compared to those showing higher *PKCα* mRNA expression. This model also confirmed that early T immunophenotype patients presented a worse outcome compared to the others, as previously reported [10]. Furthermore, final patient stratification in the AIEOP protocol was also associated with relapse risk. *PKCα* expression value at diagnosis emerged as a new informative prognostic marker that, on top of other traditional factors, is related to a variation in outcome. Moreover, an association was found between *PKCα* mRNA expression and both prednisone response and MRD stratification, the two most important parameters taken into account to determine final AIEOP stratification.

Finally, we analyzed the relevance of *PKCα* on incidence of relapse in relation to MRD strata. Low *PKCα* expression discriminated, within the HR and IR classes, a subgroup of patients with a higher incidence of relapse. In particular, the defined threshold identified patients at higher risk within the HR for MRD set, with a significantly increased risk of relapse. Thus, within the HR class, we could discriminate by *PKCα* mRNA expression a group of patients who are characterized by a very negative prognosis and who are not identified by other prognostic factors. From a clinical standpoint, it seems highly relevant to identify these patients, as they could be proposed for experimental therapeutic protocols aiming to improve their outcome. Moreover, we performed Gene Set Enrichment Analysis (GSEA) on gene expression profiles of MRD-HR patients and differences in gene expression patterns between patients with low and high *PKCα* expression levels respectively were revealed. In particular, enrichment of gene sets comprising those involved in cell cycle, telomerase maintenance and RNA transcription was observed in the MRD-HR low *PKCα* expression subgroup. This finding points to a high proliferative and more aggressive phenotype in leukemic cells of these patients that were characterized by a worse prognosis.

Recently, early T-cell precursor acute lymphoblastic leukemia (ETP-ALL) was identified as a T-ALL subgroup characterized by a very poor outcome and treatment failure [11]. Since ETP- ALL immunophenotype markers were not completely characterized in the ALL2000- ALLR2006 protocol, ETP-ALL patients were not considered in our study cohort. Analysis of a set of newly diagnosed ETP-ALL patients remarkably revealed a lower *PKCα* mRNA expression than the defined threshold (data not shown).

In this study, a novel promising prognostic biomarker in childhood T-ALL was discovered: *PKCα* is down-regulated at diagnosis in T-ALL patients with a high chance of relapse. Within the MRD-HR stratum, we identified a new very high risk subgroup of T-ALL patients characterized by low *PKCα* expression levels. *PKCα* expression at diagnosis thus proved to be a very strong predicting factor associated with relapse in pediatric T-ALL. For this reason, *PKCα* expression might be taken into account for a more precise and reliable patient stratification in order to improve treatment selection. An additional easy test to evaluate *PKCα* expression levels at diagnosis of T-ALL could refine final stratification of risk classes and bone marrow transplantation induction choice. The investigation of new experimental therapeutic approaches is needed to identify alternative cures for these pediatric T-ALL patients.

## METHODS

### Patients

Bone marrow samples from T-ALL pediatric patients at diagnosis, collected at the Pediatric Oncohematology Laboratory of Padova (Italy) between 2000 and 2010, were retrospectively studied. Diagnosis was made according to standard cytomorphology, cytochemistry, and immunophenotypic criteria [25]. Mononuclear cells from patients bone marrow were separated using the Ficoll-Hypaque technique (Pharmacia, Uppsala, Sweden). Blast percentage was comprised between 70% and 98%.

Proteome from 98 patients at diagnosis of T-ALL was studied by RPPA. These patients studied were enrolled in ALL88, ALL91, ALL95 and ALL2000, the therapeutic protocols of the Associazione Italiana Ematologia Oncologia Pediatrica (AIEOP) and of the Berlin-Frankfurt- Münster (BFM)-oriented Group (Table S1).

For *PKCα* mRNA expression analysis, a cohort of 173 T-ALL patients was selected from the cohort of 422 T-ALL patients enrolled in Italy in the AIEOP-BFM ALL2000-ALLR2006 treatment protocol and according to RNA sample availability. Based on MRD levels (standard [MRD-SR], intermediate [MRD-IR], or high risk [MRD-HR] for MRD as described by Schrappe et al. [10]), response to the first week of steroids (≥1000 circulating blasts/μL: poor prednisone response [PPR]; <1000 circulating blasts/μL: good prednisone response [PGR]) and resistance to induction therapy, patients were divided into final stratification groups (SR, IR, and HR) and treated with risk-adjusted polychemotherapeutic regimens.

The current study was approved by the relevant ethical committees and informed consent was provided by each patient's parent(s) or legal guardian(s), following the tenets of the Declaration of Helsinki.

### T cells from healthy donors

Mononuclear cells from peripheral blood (PB) of 4 healthy donors were isolated using Ficoll- Hypaque density gradient centrifugation. Healthy T-cells were isolated from PB mononuclear cells by depletion of non T-cells using human Pan T Cell Isolation Kit II (Miltenyi Biotec, Bergisch Gladbach, Germany). A Miltenyi autoMACS magnetic cell sorter (Miltenyi Biotec) was used to purify T cells and isolated cells purity was confirmed by flow cytometry. The purity of the isolated T-cells was 99%, as determined by flow cytometric analysis. T-cells were pooled and used as normal control (calibrator) in RQ-PCR experiments.

### Reverse Phase Protein Arrays (RPPA) screening and validation

Cell lysis, protein extraction and quantification, samples dilution and RPPA procedure were performed as described by Accordi et al. [26]. Protein lysates were loaded into a 384-well plate and serially diluted with dilution buffer into four-point dilution curves (from undiluted to 1:8). Two commercial cell line lysates, Jurkat and HeLa Pervanadate (BD Biosciences, Franklin Lakes, NJ), and Reh treated with doxorubicin 100nM (Amersham Biosciences) were used as positive controls for antibody staining. Samples were printed in duplicate, in a 4 points dilution curve, on nitrocellulose-coated glass slides (FAST slides, Whaterman Schleicher & Schuell, Florham Park, NJ) using the 2470 Arrayer (Aushon BioSystems, Burlington, MA). Selected slides were stained with FAST GREEN FCF (Sigma-Aldrich, St. Louis, MO) 0.01%, a fluorescent dye used to estimate the total protein amount for each printed sample, and scanned by ScanArray 4000 (Packard Biochip Technologies, Billerica, MA). Before antibody staining, slides were blocked in blocking solution (I-Block 2% - Applied BioSystems, Foster City, CA-, 0.1% Tween20 - Sigma - in PBS 1X) for 3 hours at room temperature. Then, blocked slides were stained with antibodies on an automatic slide stainer (Dako Autostaines Plus, Dako Cytomation, Carpinteria, CA) using the CSA kit (Dako Cytomation). Each RPPA slide was stained with a specific primary antibody. 53 antibodies specific for key signalling molecules involved in different cell pathways were tested (Table S2). Each primary antibody used in RPPA was previously subjected to an extensive validation for single band specificity by Western Blot [26]. The TIF images of antibody or Fast Green FCF stained slides were analyzed using MicroVigeneTM software (VigeneTech Inc, Boston, MA) and every protein expression or activation signal was quantified for each sample. RPPA data were then validated by Western Blot analyses. Total cells lysates were analyzed by SDS-PAGE under reducing conditions. 20 μg from total protein fraction were loaded in a precast gel (Criterion, Biorad, Hercules, CA) and then proteins were transferred to 0.2 mm polyvinylidene difluoride membrane (PVDF) (Hybond-P, GE Healthcare, Chalfont St. Giles, UK) following standard methods. Primary antibodies: anti – PKCα S657 (1:1000) (CellSignaling Technology, Inc, Danvers, MA); anti – PKCα (1:1000) (Millipore, Billerica, MA), anti - βActin (1:10.000) (Sigma-Aldrich). Secondary antibodies: HRP-Goat anti-rabbit and anti-mouse IgG-conuigate (1:50000) (Zymed Laboratories; Inc., South San Francisco, CA).

### RNA extraction and real-time quantitative PCR

Total RNA from each specimen was isolated using TRIZOL (Life Technologies, Carlsbad, CA) following the manufacturer's instructions. RNA quality and concentration were assessed using the Agilent 2100 Bioanalyzer (Agilent Technologies, Palo Alto, CA) and the NanoDrop ND-1000 spectrophotometer (NanoDrop Technologies Inc.) respectively.

Reverse transcription (RT)–polymerase chain reaction (PCR) was performed starting from 1 μg of total RNA. The cDNA was synthesized using SuperScriptII reverse transcriptase (Life Technologies). RNA was primed at 75°C for 10 minutes and the RT reaction was subsequently performed in a total volume of 20 μL as follows: 25°C for 10 minutes, 42°C for 30 minutes, and 99.9°C for 5 minutes.

*PKCα* expression in patients was quantified by SYBR Green Real-Time Quantitative PCR (RQ-PCR) with the Platinum SYBR Green qPCR SuperMix UDG (Life Technologies), by means of the 7900 HT Fast RQ-PCR System (Applied Biosystems, Foster City, CA). The housekeeping gene *GUSB* was chosen as a reference. The cDNA from normal T-cells was used as a calibrator. *PKCα* expression was determined using the 2-ΔΔCt method. *PKCα* primer sequences were Fwd: 5'-GCAAAGGAGCAG AGAACT-3', 5'-TACTGCACTCTGTAAGATGG-3'. *GUSB* primer sequences 5'-GAAAATATGTGGTT GGAGAGCTCATT-3', and 5'-CGAGTGAAGATCCC CTTTTTA-3'.

### Gene expression analyses

Thirteen T-ALL patients, belonging to the MRD-HR group and previously analyzed by RQ-PCR for *PKCα* expression, were studied using Gene Expression Profiling. With respect to microarray experiments in vitro transcription, hybridization and biotinin labeling were performed according to Affymetrix 3'IVT Labeling protocol. GeneChip Human Genome U133 Plus 2.0 arrays (Affymetrix, Santa Clara, CA) were used. Microarray data (CEL files) were generated using default Affymetrix microarray analysis parameters (GeneChip Command Console Software (AGCC); Affymetrix). CEL files can be found at GEO depository (GEO, http://www.ncbi.nlm.nih.gov/geo/; Series Accession Number GSE39816). All generated CEL files were normalized using robust multiarray averaging (RMA) expression measure of Affy-R package (www.r-project.org).

Gene Set Enrichment Analysis (GSEA) was performed using GSEA v2.0 (http://www.broadinstitute.org/gsea) with probes ranked by signal-to-noise ratio and statistical significance determined by 1000 gene set permutations. Gene set permutations were used to enable direct comparisons between two groups of MRD-HR T-ALL defined on *PKCα* expression according to the threshold (see “Results”, under “*PKCα* mRNA expression at diagnosis as prognostic biomarker: definition of a threshold”). Minimum gene set size was set to 15. Maximum gene set size was set to 500. We considered enriched gene sets with a false discovery rate (FDR)<0.05. The reference gene sets used in GSEA analyses are the following: gene set C2, gene sets collected from various sources such as online pathway databases, publications in PubMed and knowledge of domain experts; gene set C4, cancer gene neighbourhood GSEA MsigDB database; gene set C6, gene sets of oncogenic signatures. The relationship of clusters identified in the two MRD-HR groups defined on *PKCα* expression was established by cross GSEA analysis [27] of the top up- and down-regulated genes (Student's t test, P < 0.0001). Heatmap shows the top 50 genes that are differentially expressed between the two MRD- HR subgroups; expression values are represented as range of colours, where the range of colours shows the range of expression values.

### Statistical analyses

Overall survival (OS) and event-free survival (EFS) were calculated from the date of diagnosis to the date of event, i.e. death from any cause with respect to OS and resistance, relapse, death or second malignant neoplasm, whichever occurred first, as to EFS. OS and EFS curves were estimated using the Kaplan–Meier method; groups were compared using the log-rank test. Cumulative incidence of relapse (CIR) was estimated by adjusting for the competing risks of other events and compared using the Gray test. The Cox regression model was applied to evaluate the role of *PKCα* on the cause-specific hazard of relapse, adjusting for the main prognostic factors: final risk stratification (HR vs. IR and SR), age (≥10 vs. <10 years), white blood cell (WBC) count (>50000/mm3, ≤50000/mm3), and immunophenotype (early T vs. others). To identify the cut-off discriminating between patients at higher and lower risk of relapse, the Youden index [sensitivity + (1 - specificity)] [28] was used to maximize both specificity and sensitivity on the receiver operating characteristic (ROC) curve, after extending to time-to-event data [29]. The Chi-square test was used to evaluate the association between *PKCα* expression and the T-ALL main prognostic factors. All tests were two-sided; analyses were performed using R (http://cran.r-project.org/).

## SUPPLEMENTAL TABLES AND FIGURES


