# Supplementary material for: Low PKCa expression within the MRD-HR stratum defines a new subgroup of childhood T-ALL with very poor outcome
Source: Oncotarget. 2014 Jun 6;5(14):5234–45. doi: 10.18632/oncotarget.2062 (PMC4170630; doi:10.18632/oncotarget.2062)
Supplement: Supplementary file 1 [file oncotarget-05-5234-s001.pdf]

Low *PKCa* expression within the MRD-HR stratum defines a new subgroup of childhood T-ALL with very poor outcome

SUPPLEMENTAL TABLES

Table S1: Clinical and biological features of T-ALL patients studied by RPPA.

Characteristic	*Number*	%
Sex		
male	75	76,5
female	23	23,5
Age (years)		
<5	18	18,4
5-10	44	44,9
>10	36	36,7
AIEOP[a] therapy protocol		
ALL[b] 88	1	1
ALL 91	5	5,1
ALL 95	10	10,2
ALL 2000	82	83,7
Immunophenotype T-ALL		
early	35	35,7
thymic	42	42,9
intermediate	5	5,1
intermediate/mature	4	4,1
mature	12	12,2
WBC count		
>50000/mm^3	64	66,7
<50000/mm^3	32	33,3
ND	2	
Prednisone Response (8th day of treatment)		
good (leukemic blasts<1000/µL)	59	63,4
poor (leukemic blasts>1000/µL)	34	36,6
ND	5	
MRD (only ALL- 2000 AIEOP patients)		
standard risk	15	18,1
intermedium risk	48	57,8
high risk	20	24,1
ND	15	
Relapse		
yes	30	31,3
no	66	68,7
ND	2	
DNA Index		
1-1.16	82	97,6
>1.16	2	2,4
ND[c]	14	
CNS invasion		
yes	10	10,3
no	87	89,7
ND	1	
Outcome		
dead	29	29,6
CR[d]	69	70,4
del1p32		
yes	7	8,5
no	75	91,5
ND	16	
t(10;11)		
yes	7	12,1
no	51	87,9
ND	40	

[a]AIEOP= Associazione Italiana Ematologia Oncologia Pediatrica.
[b]ALL= Acute Lymphoblastic Leukemia.
[c]ND= No Data.
[d]CR= Complete Remission

Table S2: Antibodies used in T-ALL RPPA study.

Antibody	Catalog number #	MW (kDa)	Company
4E-BP1 (S65)	9451	15-20	CellSignaling
Akt (S473)	9271	60	CellSignaling
Akt (T308)	9275	60	CellSignaling
Annexin II	610068	36	BD
A-Raf (S299)	4431	68	CellSignaling
Bak	06-536	30	Upstate
Bax	2772	20	CellSignaling
Bcl-2 (S70)	2827	28	CellSignaling
Bcl-2 (T56)	2875	28	CellSignaling
Bcl-xL	2762	30	CellSignaling
B-Raf (S445)	2696	95	CellSignaling
Caspase-6, cleaved (D162)	9671	18	CellSignaling
Caspase-7, cleaved (D198)	9491	20	CellSignaling
Caspase-9, cleaved (D330)	9501	17, 37	CellSignaling
CDK2 (78B2)	2546	33	CellSignaling
Cleaved NOTCH1 Val1744	2421	110	CellSignaling
CREB (S133)	9191	43	CellSignaling
Cyclin A clone BF683	05-374	58	Upstate
Cyclin B	610220	62	BD
Cyclin D1 (G124-326)	554180	36	BD
Cyclin E (HE12)	554182	50	BD
eNOS/NOS III (S116)	07-357	132	Upstate
ERK 1/2 (T202/Y204)	9101	42, 44	CellSignaling
FADD (S194)	2781	28	CellSignaling
FAK (Y397) (18)	611806	125	BD
GRB2	3972	25	CellSignaling
GSK-3alpha/beta (S21/9)	9331	46, 51	CellSignaling
HSP70 (C92F3A-5)	SPA-810	70	Stressgen
IRS-1 (S612)	2386	180	CellSignaling
JAK1 (Y1022/1023)	3331	130	CellSignaling
KIP1/p27 (57)	610241	27	BD
LCK (Y505)	2751	56	CellSignaling
MEK1/2 (S217/221)	9121	45	CellSignaling
mTOR (S2448)	2971	289	CellSignaling
NF-kappaB p65 (S536)	3031	65	CellSignaling
p38 MAP KINASE (T180/Y182)	9211	40	CellSignaling
p70 S6 KINASE (T389)	9205	70, 85	CellSignaling
p90RSK (S380)	9341	90	CellSignaling
PARP, cleaved (D214)	9541	89	CellSignaling
PDK1 (S241)	3061	63	CellSignaling
PKA C (T197)	4781	42	CellSignaling
PKC alpha (S657)	06-822	82	Upstate
PKC alpha/beta II (T638/641)	9375	80, 82	CellSignaling
PKC delta (T505)	9374	78	CellSignaling
PKC theta (T538)	9377	79	CellSignaling
PTEN (S380)	9551	54	CellSignaling
S6 RIBOSOMAL PROTEIN (S235/236)	4856	32	CellSignaling
SAPK/JNK (T183/Y185)	9251	46, 54	CellSignaling
SMAC/DIABLO	2954	21	CellSignaling
STAT1 (Y701)	9171	84, 91	CellSignaling
STAT3 (S727)	9134	79, 86	CellSignaling
STAT5 (Y694)	9351	90	CellSignaling
ZAP-70 (Y319)/ SYK (Y352)	2717	70, 72	CellSignaling

Tables S3 - S4 - S5:

Gene sets differentially expressed between the two MRD-HR groups of patients, categorized on *PKCα* defined threshold, are reported in Table S4 (gene set C2), Table S5 (gene set C4) and Table S6 (gene set C6) (FDR< 0.05). Abbreviations: Name, Gene Set Name; Size, number of genes in the gene set after filtering out those genes not in the expression dataset; ES, Enrichment Score for gene set (the degree to which this gene set is overrepresented at the top or bottom of the ranked list of genes in the expression dataset); NES, Normalized Enrichment Score (the enrichment score for the gene set after it has been normalized across analyzed gene sets); NOM p-val, Nominal p-value (the statistical significance of the enrichment score); FDR q-val, False Discovery rate (the estimated probability that the normalized enrichment score represents a false positive finding); Leading Edge, displaying the three statistics used to define the leading edge subset: Tags, the percentage of gene hits before (for positive ES) or after (for negative ES) the peak in the running enrichment score to give an indication of the percentage of genes contributing to the enrichment score. List, the percentage of genes in the ranked gene list before (for positive ES) or after (for negative ES) the peak in the running enrichment score to give an indication of where the enrichment score is attained in the list; signal, the enrichment signal strength.

Table S3

NAME	SIZE	ES	NES	NOM p-val	FDR q-val	LEADING EDGE
REACTOME_MEIOTIC_RECOMBINATION	70	-0.80	-3.00	0.000	0.000	tags=53%, list=6%, signal=56%
REACTOME_RNA_POL_I_PROMOTER_OPENING	47	-0.87	-2.98	0.000	0.000	tags=66%, list=6%, signal=70%
REACTOME_RNA_POL_I_TRANSCRIPTION	70	-0.80	-2.97	0.000	0.000	tags=50%, list=6%, signal=53%
REACTOME_MEIOSIS	96	-0.74	-2.96	0.000	0.000	tags=43%, list=6%, signal=45%
REACTOME_PACKAGING_OF_TELOMERE_ENDS	39	-0.87	-2.94	0.000	0.000	tags=67%, list=6%, signal=71%
REACTOME_DEPOSITION_OF_NEW_CENPA_CONTAINING_NUCLEOSOMES_AT_THE_CENTROMERE	50	-0.84	-2.93	0.000	0.000	tags=64%, list=7%, signal=68%
REACTOME_CHROMOSOME_MAINTENANCE	100	-0.73	-2.90	0.000	0.000	tags=48%, list=13%, signal=55%
REACTOME_AMYLOIDS	66	-0.78	-2.86	0.000	0.000	tags=48%, list=6%, signal=51%
REACTOME_MEIOTIC_SYNAPSIS	60	-0.79	-2.85	0.000	0.000	tags=52%, list=7%, signal=56%
REACTOME_RNA_POL_I_RNA_POL_III_AND_MITOCHONDRIAL_TRANSCRIPTION	103	-0.71	-2.82	0.000	0.000	tags=37%, list=6%, signal=39%
REACTOME_TELOMERE_MAINTENANCE	62	-0.77	-2.74	0.000	0.000	tags=45%, list=7%, signal=49%
REACTOME_CELL_CYCLE	345	-0.56	-2.56	0.000	0.000	tags=38%, list=17%, signal=45%
REACTOME_TRANSCRIPTION	178	-0.56	-2.41	0.000	0.000	tags=23%, list=6%, signal=24%
REACTOME_MITOTIC_PROMETAPHASE	74	-0.61	-2.24	0.000	0.000	tags=50%, list=21%, signal=63%
REACTOME_MITOTIC_M_M_G1_PHASES	147	-0.51	-2.14	0.000	0.000	tags=44%, list=25%, signal=59%
REACTOME_CELL_CYCLE_MITOTIC	271	-0.46	-2.08	0.000	0.000	tags=32%, list=17%, signal=38%
REACTOME_G2_M_CHECKPOINTS	32	-0.65	-2.04	0.000	0.001	tags=53%, list=18%, signal=65%
REACTOME_KINESINS	22	-0.70	-2.04	0.000	0.001	tags=55%, list=16%, signal=65%
REACTOME_DNA_REPLICATION	166	-0.48	-2.01	0.000	0.001	tags=43%, list=25%, signal=57%
REACTOME_MITOTIC_G2_G2_M_PHASES	69	-0.52	-1.98	0.000	0.002	tags=35%, list=14%, signal=40%
REACTOME_RECRUITMENT_OF_MITOTIC_CENTROSOME_PROTEINS_AND_COMPLEXES	54	-0.55	-1.94	0.000	0.004	tags=37%, list=14%, signal=43%
REACTOME_LOSS_OF_NLP_FROM_MITOTIC_CENTROSOMES	47	-0.55	-1.90	0.000	0.007	tags=38%, list=14%, signal=44%
REACTOME_ACTIVATION_OF_ATR_IN_RESPONSE_TO_REPLICATION_STRESS	27	-0.60	-1.86	0.000	0.011	tags=59%, list=25%, signal=79%
REACTOME_FACTORS_INVOLVED_IN_MEGAKARYOCYTE_DEVELOPMENT_AND_PLATELET_PRODUCTION	117	-0.44	-1.78	0.000	0.025	tags=30%, list=16%, signal=35%
REACTOME_ACTIVATION_OF_THE_PRE_REPLICATIVE_COMPLEX	22	-0.60	-1.73	0.006	0.040	tags=77%, list=34%, signal=117%
REACTOME_SEMA4D_INDUCED_CELL_MIGRATION_AND_GROWTH_CONE_COLLAPSE	23	-0.58	-1.72	0.008	0.042	tags=22%, list=9%, signal=24%
REACTOME_FANCONI_ANEMIA_PATHWAY	19	-0.61	-1.72	0.016	0.042	tags=47%, list=17%, signal=57%
REACTOME_DOUBLE_STRAND_BREAK_REPAIR	22	-0.59	-1.72	0.008	0.041	tags=77%, list=33%, signal=115%
REACTOME_CELL_DEATH_SIGNALLING_VIA_NRAGE_NRIF_AND_NADE	56	-0.48	-1.70	0.000	0.049	tags=48%, list=29%, signal=68%

Table S4

NAME	SIZE	ES	NES	NOM p-val	FDR q-val	LEADING EDGE
GNF2_CDC20	50	-0.80	-2.79	0.000	0.000	tags=80%, list=15%, signal=94%
GNF2_CCNB2	51	-0.79	-2.79	0.000	0.000	tags=78%, list=15%, signal=92%
GNF2_CDC2	54	-0.77	-2.74	0.000	0.000	tags=74%, list=15%, signal=87%
GNF2_CCNA2	61	-0.76	-2.74	0.000	0.000	tags=72%, list=15%, signal=85%
GNF2_HMMR	43	-0.80	-2.68	0.000	0.000	tags=77%, list=14%, signal=89%
GNF2_CENPF	54	-0.74	-2.63	0.000	0.000	tags=69%, list=15%, signal=81%
GNF2_PCNA	61	-0.72	-2.62	0.000	0.000	tags=66%, list=16%, signal=78%
GNF2_ESPL1	35	-0.81	-2.62	0.000	0.000	tags=69%, list=9%, signal=75%
GNF2_RRM1	80	-0.68	-2.61	0.000	0.000	tags=61%, list=20%, signal=76%
GNF2_CENPE	36	-0.80	-2.58	0.000	0.000	tags=78%, list=14%, signal=90%
GNF2_MCM4	50	-0.72	-2.54	0.000	0.000	tags=62%, list=15%, signal=73%
GNF2_RRM2	36	-0.77	-2.53	0.000	0.000	tags=72%, list=15%, signal=85%
GNF2_SMC4L1	74	-0.68	-2.52	0.000	0.000	tags=65%, list=23%, signal=84%
GNF2_BUB1B	46	-0.74	-2.52	0.000	0.000	tags=65%, list=14%, signal=76%
GNF2_BUB1	25	-0.84	-2.51	0.000	0.000	tags=68%, list=9%, signal=74%
GNF2_SMC2L1	29	-0.81	-2.50	0.000	0.000	tags=79%, list=13%, signal=91%
GNF2_CKS2	45	-0.74	-2.49	0.000	0.000	tags=62%, list=13%, signal=72%
GNF2_MKI67	25	-0.84	-2.49	0.000	0.000	tags=92%, list=15%, signal=108%
GNF2_TTK	34	-0.75	-2.44	0.000	0.000	tags=74%, list=18%, signal=89%
MODULE_54	237	-0.54	-2.38	0.000	0.000	tags=49%, list=20%, signal=61%
GNF2_RFC4	57	-0.67	-2.36	0.000	0.000	tags=53%, list=15%, signal=62%
GNF2_CKS1B	36	-0.73	-2.36	0.000	0.000	tags=72%, list=20%, signal=90%
GNF2_FEN1	48	-0.67	-2.30	0.000	0.000	tags=54%, list=16%, signal=64%
GNF2_H2AFX	27	-0.74	-2.29	0.000	0.000	tags=78%, list=20%, signal=97%
MORF_BUB1B	61	-0.61	-2.28	0.000	0.000	tags=41%, list=15%, signal=48%
MODULE_222	22	-0.75	-2.21	0.000	0.000	tags=50%, list=6%, signal=53%
GNF2_RFC3	38	-0.67	-2.20	0.000	0.000	tags=50%, list=14%, signal=58%
MODULE_168	22	-0.74	-2.19	0.000	0.000	tags=50%, list=6%, signal=53%
MODULE_189	21	-0.74	-2.13	0.000	0.000	tags=62%, list=12%, signal=71%
MODULE_90	16	-0.78	-2.12	0.000	0.000	tags=56%, list=9%, signal=62%
MODULE_127	24	-0.71	-2.09	0.000	0.000	tags=54%, list=12%, signal=62%
MORF_BUB1	48	-0.59	-2.08	0.000	0.000	tags=58%, list=29%, signal=82%
MODULE_534	15	-0.77	-2.07	0.000	0.000	tags=60%, list=10%, signal=67%
MORF_CCNF	63	-0.56	-2.06	0.000	0.000	tags=40%, list=15%, signal=47%
MODULE_552	17	-0.78	-2.04	0.000	0.001	tags=65%, list=12%, signal=74%
MODULE_421	25	-0.69	-2.03	0.000	0.001	tags=56%, list=13%, signal=64%
MODULE_158	39	-0.61	-1.99	0.000	0.001	tags=64%, list=30%, signal=91%
MODULE_403	43	-0.59	-1.98	0.000	0.002	tags=37%, list=16%, signal=44%
GNF2_TNFRSF1B	58	-0.55	-1.96	0.000	0.002	tags=41%, list=15%, signal=49%
GNF2_HCK	87	-0.50	-1.95	0.000	0.002	tags=45%, list=18%, signal=55%
MODULE_125	40	-0.59	-1.94	0.002	0.003	tags=40%, list=15%, signal=47%
GNF2_SPI1	34	-0.59	-1.93	0.000	0.003	tags=62%, list=17%, signal=75%
GNF2_TNFSF10	30	-0.61	-1.93	0.000	0.003	tags=63%, list=18%, signal=77%
MODULE_303	27	-0.61	-1.92	0.000	0.004	tags=52%, list=22%, signal=66%
GNF2_CARD15	61	-0.53	-1.91	0.000	0.004	tags=33%, list=9%, signal=36%
MODULE_57	51	-0.53	-1.88	0.000	0.006	tags=39%, list=15%, signal=46%
MODULE_320	18	-0.68	-1.88	0.000	0.006	tags=56%, list=11%, signal=62%
MODULE_315	15	-0.72	-1.87	0.000	0.006	tags=53%, list=5%, signal=56%
GNF2_PECAM1	49	-0.53	-1.86	0.002	0.006	tags=45%, list=20%, signal=56%
GNF2_MSH6	30	-0.60	-1.86	0.000	0.007	tags=63%, list=29%, signal=89%
GNF2_CD1D	40	-0.55	-1.84	0.002	0.009	tags=35%, list=9%, signal=38%
MODULE_451	26	-0.61	-1.84	0.002	0.009	tags=35%, list=6%, signal=37%
GNF2_MSH2	25	-0.62	-1.83	0.002	0.010	tags=44%, list=18%, signal=54%
MORF_RRM1	89	-0.47	-1.83	0.000	0.010	tags=51%, list=31%, signal=73%
GNF2_ATM	26	-0.62	-1.82	0.000	0.010	tags=54%, list=17%, signal=65%
GNF2_S100A4	42	-0.55	-1.82	0.004	0.010	tags=52%, list=19%, signal=65%
MODULE_204	79	-0.47	-1.79	0.000	0.015	tags=34%, list=15%, signal=40%
GNF2_MCM5	50	-0.51	-1.77	0.002	0.017	tags=44%, list=25%, signal=59%
MODULE_252	213	-0.40	-1.77	0.000	0.017	tags=37%, list=23%, signal=47%
MODULE_124	86	-0.45	-1.76	0.002	0.019	tags=30%, list=14%, signal=35%
MORF_DNMT1	104	-0.43	-1.75	0.000	0.021	tags=47%, list=31%, signal=68%
MODULE_198	274	-0.38	-1.73	0.000	0.025	tags=32%, list=20%, signal=40%
MODULE_244	174	-0.40	-1.70	0.000	0.030	tags=37%, list=23%, signal=48%
MORF_RFC4	134	-0.42	-1.70	0.000	0.031	tags=37%, list=26%, signal=49%
MORF_ESPL1	58	-0.47	-1.69	0.000	0.034	tags=48%, list=31%, signal=69%
GNF2_PA2G4	72	-0.45	-1.68	0.000	0.035	tags=50%, list=30%, signal=72%

Table S5

NAME	SIZE	ES	NES	NOM p-val	FDR q-val	LEADING EDGE
JAK2_DN.V1_DN	132	-0.45	-1.87	0.000	0.007	tags=37%, list=20%, signal=46%
STK33_SKM_UP	249	-0.41	-1.84	0.000	0.006	tags=26%, list=10%, signal=28%
PRC2_EZH2_UP.V1_UP	170	-0.42	-1.77	0.000	0.009	tags=29%, list=14%, signal=34%
EGFR_UP.V1_DN	174	-0.38	-1.63	0.000	0.031	tags=33%, list=17%, signal=39%
GCNP_SHH_UP_EARLY.V1_UP	149	-0.38	-1.60	0.000	0.034	tags=28%, list=18%, signal=34%
VEGF_A_UP.V1_DN	174	-0.37	-1.59	0.002	0.033	tags=32%, list=15%, signal=37%
RB_P107_DN.V1_UP	125	-0.39	-1.59	0.002	0.029	tags=26%, list=13%, signal=29%
RPS14_DN.V1_DN	157	-0.37	-1.56	0.002	0.036	tags=30%, list=15%, signal=35%
MTOR_UP.N4.V1_UP	186	-0.36	-1.54	0.000	0.041	tags=28%, list=14%, signal=32%
BCAT_BILD_ET_AL_DN	41	-0.45	-1.53	0.019	0.037	tags=32%, list=19%, signal=39%
PRC2_EDD_UP.V1_UP	171	-0.36	-1.53	0.002	0.034	tags=30%, list=17%, signal=36%

SUPPLEMENTAL FIGURES

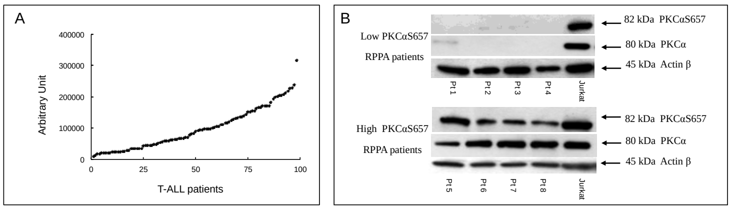

Figure S1: RPPA PKCαS657 activation values in T-ALL patients (A). Validation of RPPA results and PKCα total protein analysis (B). PKCαS657 activation level was confirmed by Western Blot analyses in a group of T-ALL patients, with high and low PKCαS657 activation, previously studied by phosphoproteomic analysis. PKCα total protein was also analyzed.

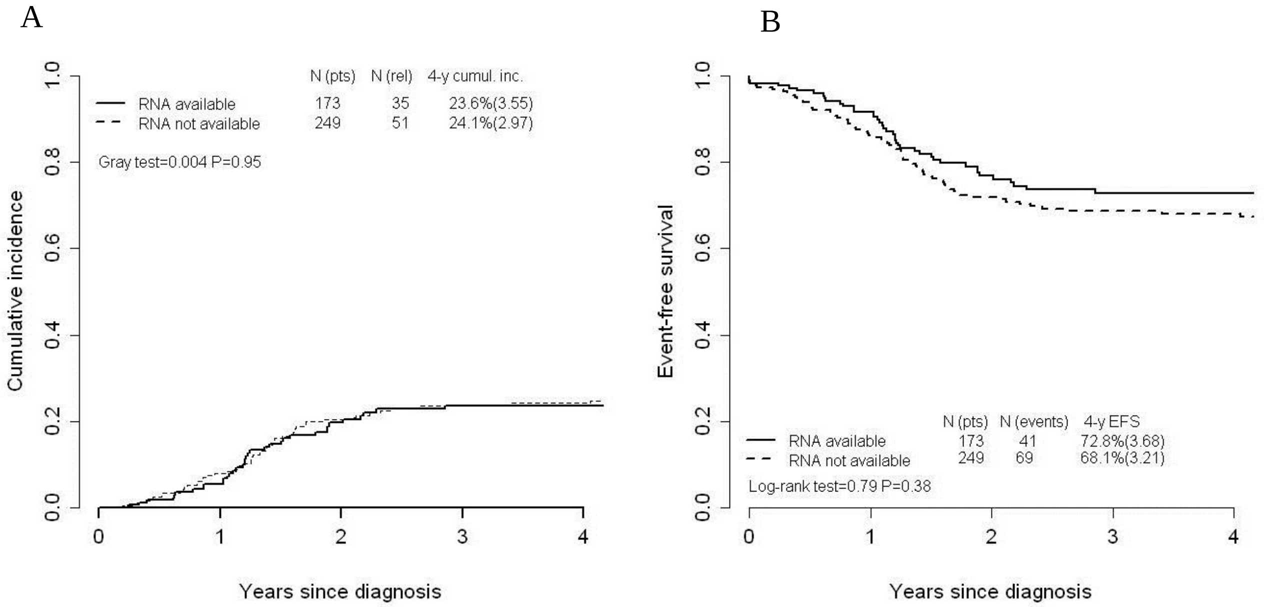

Figure S2: Cumulative incidence of relapse (A) and event-free survival (B) in the ALL2000-ALLR2006 AIEOP treatment protocol cohort. Comparison of relapse incidence and event-free survival between the T-ALL group of patients included in the *PKCα* expression RQ-PCR study (RNA available) and the T-ALL cases not included in the study (RNA not available).

Abbreviations: Cumul.inc., cumulative incidence; EFS, event-free survival; pts, patients; rel, relapse; T-ALL, T-cell acute lymphoblastic leukemia.

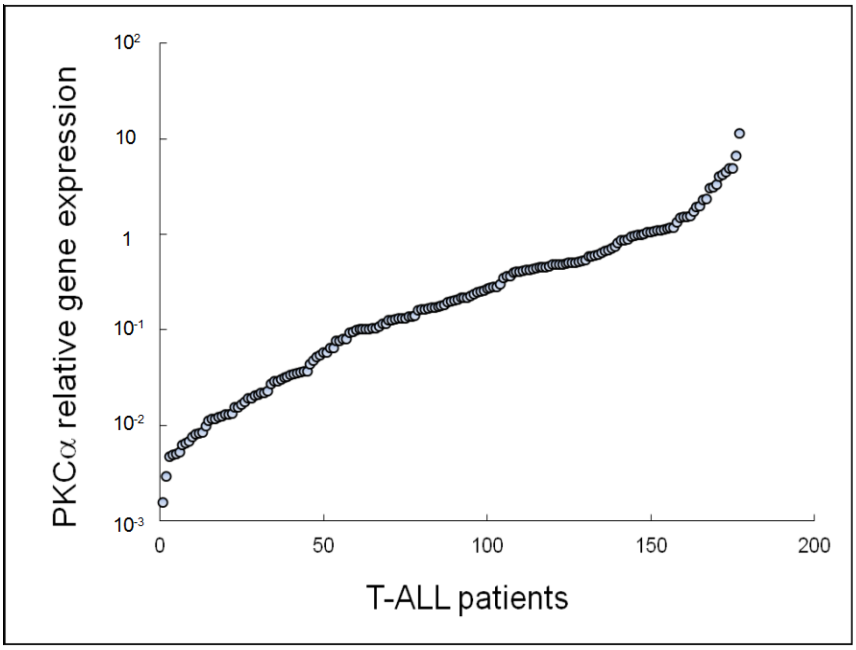

Figure S3: RQ-PCR *PKCα* gene expression values in the AIEOP_BFM ALL2000-ALLR2006 AIEOP protocol analyzed cohort. Abbreviation: T-ALL, T-cell acute lymphoblastic leukemia.

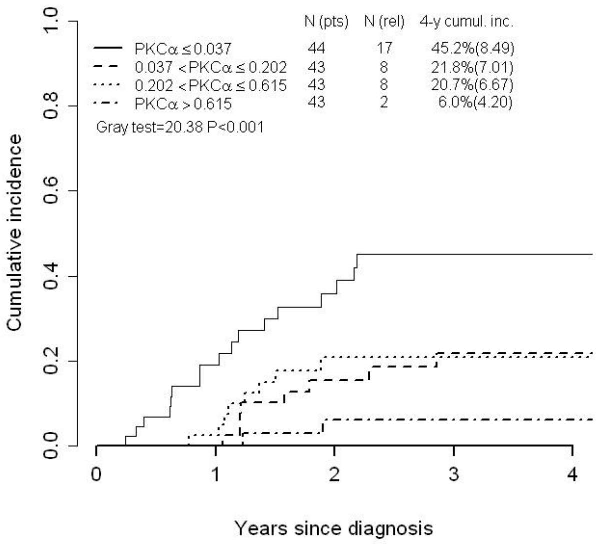

Figure S4: Cumulative incidence of relapse in T-ALL patients studied by RQ-PCR. Patients were divided into four groups on the basis of the quartiles defined using *PKCα* mRNA expression values (1st quartile=0.037; 2nd quartile/median=0.202; 3rd quartile=0.615). Groups defined based on *PKCα* values showed the following percentages of relapse: 45.2% of T-ALL patients under the 25th percentile (N=44) relapsed whereas only 6% of the patients whose *PKCα* transcript level was higher than the 75th percentile (N=43), encountered relapse (4-year relapse cumulative incidence).

Abbreviations: Cumul.inc., cumulative incidence; pts, patients; rel, relapse; RQ-PCR, real-time quantitative polymerase chain reaction; T-ALL, T-cell acute lymphoblastic leukemia.

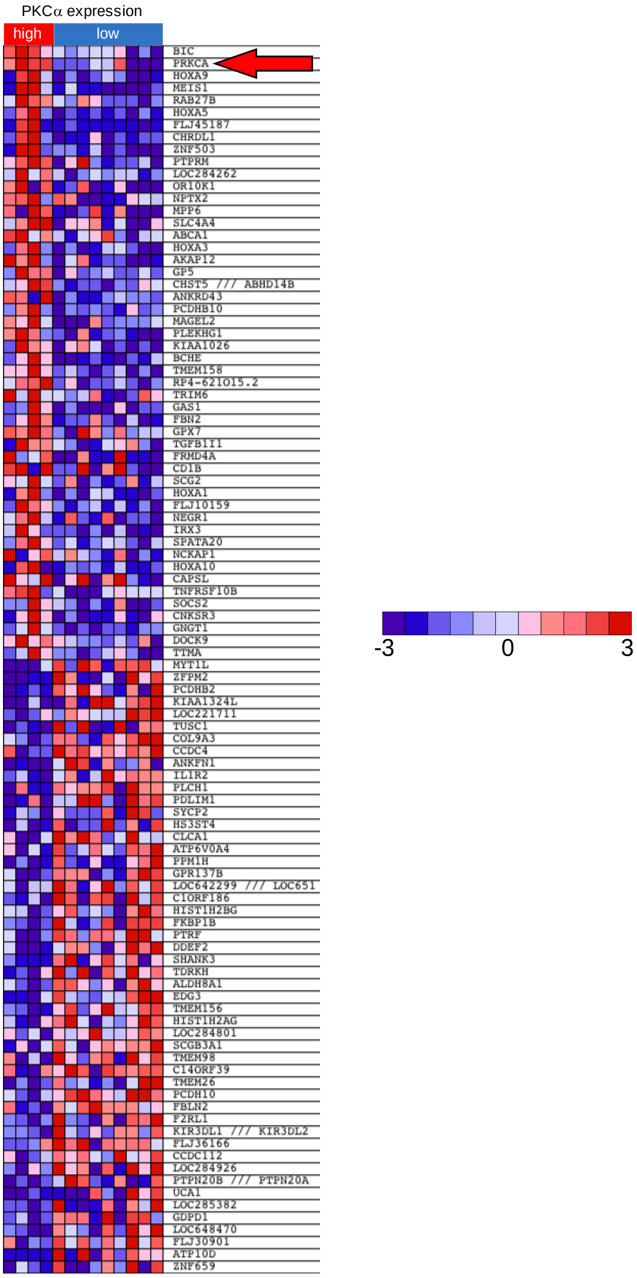

Figure S5: Heatmap of the top 50 genes differentially expressed between MRD-HR patients with high *PKCα* expression (red line) and those with low *PKCα* expression (blue line) categorized on the threshold defined by RQ-PCR analysis (FDR<0.05). Genes are shown in rows and each sample is shown in one column. The scale bar shows colour-coded differential expression from the mean in standard deviation units, with red indicating higher levels and blue lower levels of expression.

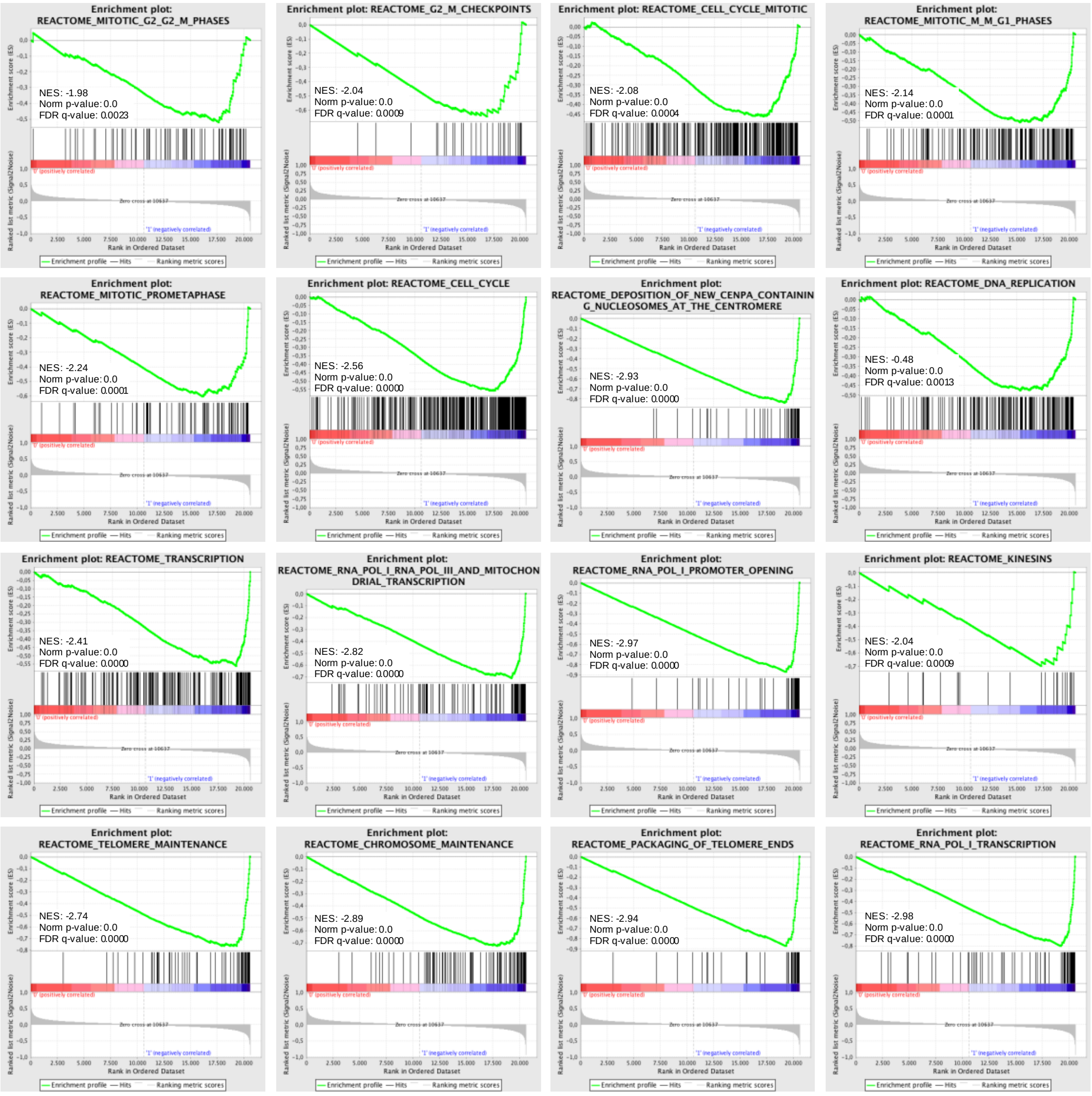

Figure S6: GSEA plots showed the enrichment of several signatures in the low *PKCα* expression MRD-HR group such as those related to cell cycle, transcription and telomerase maintenance. Negative enrichment score indicated correlation with the low *PKCα* expression MRD-HR group. Abbreviations: NES, Normalized Enrichment Score; Norm p-value, Normal p- value; FDR q-value, False Discovery Rate q-value.
